# Transfer learning of neural operators for partial differential equations based on sparse network λ-FNO

**DOI:** 10.1371/journal.pone.0321154

**Published:** 2025-05-22

**Authors:** Jinghong Xu, Yuqian Zhou, Qian Liu, Kebing Li, Haolin Yang

**Affiliations:** 1 College of Applied Mathematics, Chengdu University of Information Technology, Chengdu, Sichuan, P. R. China; 2 College of Mathematics, Southwest Minzu University, Chengdu, Sichuan, P. R. China; 3 Business School of CDUT, Chengdu University of Technology, Chengdu, Sichuan, P. R. China; Khalifa University of Science and Technology, UNITED ARAB EMIRATES

## Abstract

When the solution domain, internal parameters, and initial and boundary conditions of partial differential equation (PDE) are changed, many potential characteristics of the equation’s solutions are still similar. This provides the possibility to reduce the cost of PDE operator learning through transfer learning methods. Based on Fourier neural operator (FNO), we propose a novel sparse neural operator network named λ-FNO. By introducing the λ parameter matrix and using a new pruning method to make the network sparse, the operator learning ability of λ-FNO is greatly improved. Using λ-FNO can efficiently learn the operator from the discrete initial function space on the uniform grid to the discrete equation’s solution space on the unstructured grid, which is not available in FNO. Finally, we apply λ-FNO to several specific transfer tasks of partial differential equations under conditional distributions to demonstrate its excellent transferability. The experimental results show that when the shape of the solution domain of the equation or its internal parameters change, our framework can capture the potential invariant information of its solution and complete related transfer learning tasks with less cost, higher accuracy, and faster speed. In addition, the sparse framework has excellent extension and can be easily extended to other network architectures to enhance its performance. Our model and data generation code can get through https://github.com/Xumouren12/TL-FNO.

## 1. Introduction

Many practical problems involve solving complex partial differential equations (PDE). Examples include spacecraft design, turbulence simulation, climate prediction, oil field development, etc., and most of the complex differential equations cannot find analytical solutions, so we can only find their numerical solutions. Traditional numerical solvers commonly include finite element methods (FEM) and finite difference methods (FDM). These two methods discretize the solution domain into a finite number of grids to solve the equation. In general, the accuracy of the solution is positively related to the resolution. The higher the resolution, the higher the accuracy, but the efficiency will be lower. As the dimension of the equation increases, the total number of grid points required for discretization grows exponentially, leading to the “curse of dimensionality”, which often overwhelms traditional numerical solvers. Moreover, these traditional numerical methods are “data agnostic” [1] and are not designed to learn from available large datasets generated through simulation or observation. This limits the practical application range of traditional numerical solvers. Therefore, with the improvement of open source deep learning (DL) frameworks (such as Pytorch [2], Tensorflow [3]) in recent years and the great achievements of deep learning in many fields such as image processing [4–6], object detection [7–8], and natural language processing [9–11], people have shown great interest in solving partial differential equations through data-driven deep learning methods.

M. Raissi proposed the physical neural network (PINN) [12] in 2019. The PINNs method [13–16] uses neural networks composed of a combination of linear transformations and nonlinear activation functions and imposes physical constraints to approximate a specific solution function of the PDE. However, when a parameter or initial condition of the PDE changes slightly, the solution function will change accordingly, and we need to change and retrain the network. That limits its application to practical problems since many problems in science involve solving the same PDE system with different parameters or initial values. With the proposal of DeepONet [17] and neural operator, such as Fourier Neural Operator (FNO) [18], Multiwavelets Neural Operator [19–20], Graph Neural Operator [21], Multipole Neural Operator [22], DNO [23] and other neural operator methods [24–26], this problem has been solved to some extent. Li [27] proposed that neural operator methods are the only known models that have both discretization-invariance and universal approximation. Among the neural operator methods, FNO has been widely used for its fast speed and high precision, such as soliton mapping [28], heterogeneous material [29], CO_2_ geological storage prediction [30], and more. FNO uses neural networks to accurately approximate any nonlinear continuous operator, learning the mapping between two infinite-dimensional Banach spaces. When solving PDE, FNO learns the mapping from parameter function space or initial function space to solution function space. Therefore, changes in parameters or initial values do not require retraining of the network, which has higher application values. After we successfully learn a solution operator for a PDE model through FNO, if the initial conditions and boundary conditions of the PDE model change, since the FNO method is a data-driven method, we need to rebuild or collect a large amount of labeled data to train a new FNO operator from scratch. However, in some practical situations, collecting sufficiently large and accurate data is costly, and retraining the model is also very time-consuming.

This problem may be solved with minimal cost by the transfer learning (TL) method. In fact, Karniadakis [31] proposed a novel idea in 2022. By combining transfer learning and DeepONet, they applied the approach of transfer learning to the field of operator learning and achieved exciting results. For a PDE model, even if the PDE model undergoes some changes, the global features may be interlinked and shareable. When the network has successfully learned a solution operator for a PDE model, we can obtain the solution operator of the changed PDE model with the least cost through the TL method. TL aims to use the existing data, models, and knowledge on a specific domain (source domain) to transfer information to other different but related domains with fewer data (target domain) through domain similarity. At present, one of the more commonly used methods is the model pre-training transfer learning method. If there is already a model $f_{s}$ trained on the source domain and the target domain has few labeled data for learning, then it can apply $f_{s}$ directly on the target domain for fine-tuning. This Pre-training and fine-tuning mode do not need to train the network from scratch for new tasks, which saves time and costs, and can make our model more robust and generalize better. In the pre-training fine-tuning mode, the lower bound of the downstream transfer task is tightly dependent on the performance of pre-training [32]. The pre-training model can be used as the benchmark model for subsequent tasks. Therefore, further improving the accuracy of pre-trained models is an important challenge. Our contributions can be summarized as follows:

- We propose a novel sparse neural operator network called λ-FNO. Compared with FNO, λ-FNO can learn the operator from the discrete initial function space on the uniform grid to the discrete equation solution space on the unstructured grid, and the prediction accuracy is greatly improved when the inference time of the model is almost the same. This new architecture effectively improves the lower bound of accuracy for downstream transfer tasks.
- We combine λ-FNO, the new pruning method and the transfer learning method to obtain the transfer learning framework TL-λFNO. TL-λFNO can quickly and effectively complete specific transfer learning tasks.
- We performed the main experiments in the references [31] using TL-λFNO. The results show that TL-λFNO can solve various transfer learning problems (including changes in the domain geometry of solution, material properties, etc.) faster and more efficiently.

## 2. The λ-FNO architecture

Let $D\subset {\mathbb{R}}^{𝒹}$ be a bounded spacial domain. The spaces ${ℳ}_{1}={ℳ}_{1}(D;{\mathbb{R}}^{{𝒹}_{𝒶}}),{𝒹}_{𝒶}\in \mathbb{N}$ and ${ℳ}_{2}={ℳ}_{2}(D;{\mathbb{R}}^{{𝒹}_{𝓊}}),{𝒹}_{𝓊}\in \mathbb{N}$, denote the input function space (initial function or parametric function) and solution function space respectively. We focus on learning a nonlinear mapping $G:{ℳ}_{1}\to {ℳ}_{2},\;a\to u:=G(a)$, where the $a\in {ℳ}_{1}$, is a input function $a:D\to {\mathbb{R}}^{{𝒹}_{𝒶}}$, and the $u\in {ℳ}_{2}$, is a solution function $u:D\to {\mathbb{R}}^{{𝒹}_{𝓊}}$. Next, we construct a parametric map ${𝒢}_{\theta }:{ℳ}_{1}\times \Theta \to {ℳ}_{2},\theta \in \Theta$, to approximate 𝒢. The Θ is a parameter space. Then by minimizing the loss function L(θ) to find the optimal set of parameters $\theta^{*}$ such that ${𝒢}_{\theta^{*}}$ approximates 𝒢 best. The ℒ(θ) is defined in references [18]:


$$ℒ(\theta :={𝔼}_{a\sim {ℳ}_{1}}\left[\frac{{\left\|{𝒢}_{\theta }(𝒶)-u\right\|}^{2}}{{\left\|u\right\|}^{2}}\right]$$

we choose the Euclidean norm as the ${\left\|\cdot \right\|}^{2}$.

Then we use neural operator as the parametric map ${𝒢}_{\theta }:{ℳ}_{1}\times \Theta \to {ℳ}_{2}$ to approximate 𝒢. The neural operator was defined in references [33], neural operators are iterative architectures:


$${𝒢}_{\theta }(𝒶mathcal=Q\circ {𝒩}_{T}\circ {𝒩}_{T-1}\circ \cdot \cdot \cdot \circ {𝒩}_{0}\circ P(a)$$

where the local transformer $P:{ℳ}_{1}(D;{\mathbb{R}}^{{𝒹}_{𝒶}})\to {ℳ}_{2}(D;{\mathbb{R}}^{{𝒹}_{𝓋}})$ is a lifting operator which parameterized by a fully connected layer with activation function σ. It lifts 𝒶 to a higher-dimension function $v_{0}$. The transformer $Q:{ℳ}_{2}(D;{\mathbb{R}}^{{𝒹}_{𝓋}})\to {ℳ}_{2}(D;{\mathbb{R}}^{{𝒹}_{𝓊}})$ let the higher-dimension function back to the lower. After the transformer 𝒫, with the help of ${𝒩}_{t}:{ℳ}_{2}(D;{\mathbb{R}}^{{𝒹}_{𝓋}})\to {ℳ}_{2}(D;{\mathbb{R}}^{{𝒹}_{𝓋}})$, $v_{0}$ enters an iterative process. In the neural network, the non-linear operator layer ${𝒩}_{t}:v_{t}\to v_{t+1}$ is refer to article [18]. In our paper, we modify the neural operator iteration formula to facilitate the construction of our new framework λ-FNO. It should be noted that we introduce the λ parameter. We define the non-linear layer which makes $v_{t}\to v_{t+1}$ as follows:


$$v_{t+1}(x)={𝒩}_{t}(\lambda_{t}\circ V_{t})(x)=W_{t}\sigma {(\lambda }_{t}{\circ V}_{t})(x)+b_{t}+(𝒦(a;\theta_{t})\sigma (\lambda_{t}\circ V_{t}))(x)\forall x\in D$$ (1)

where the vector $\lambda_{t}=\left[\lambda_{0}^{\left(t\right)},\lambda_{1}^{\left(t\right)}\cdots \lambda_{t-2}^{\left(t\right)},\lambda_{t-1}^{\left(t\right)},1\right]$ and $V_{t}=[v_{0}{,v_{1}\cdots v_{t-2},v_{t-1},v_{t}]}^{T}$. Next, we discuss how to determine the data type and size of λ parameter in (1). Assume that the resolution of our two-dimensional input function discretization is H×W. For the N discretized input function $𝒶(x,t)\in {\mathbb{R}}^{H\times W}$ with the initial channel $C_{a}$, where the $C_{a}$ represents the dimension of the function and ${\mathbb{R}}^{H\times W}$ represents a set of real matrices of size H×W. So, the size of input data is $N\times C_{a}\times H\times W$. We define λ as a parameter matrix as follows:


$$\lambda =(\xi_{ij}{)}_{H\times W}\in {\mathbb{R}}^{H\times W}$$ (2)

We define the multiplication operation between vector $\lambda_{t}$ and $V_{t}$ in (1) as:


$${(\lambda }_{t}{\circ V}_{t})=v_{t}+\sum_{i=0}^{t-1}\lambda_{i}^{\left(t\right)}\cdot v_{i}.$$

$W_{t}$ and $b_{t}$ in (1) are the weight and bias parameters of this non-linear layer, respectively. The σ in the formula represents the nonlinear activation function which can improve the nonlinear fitting ability and the expression ability of characteristics. 𝒦(a;θ) is the kernel integral operator parameterized by a neural network, defined in references [18] as follows:


(𝒦(a;θ)v)(x):=∫Dk(x,y,a(x),a(y);θ)v(y)dy,∀x∈D.

**Fig 1:**
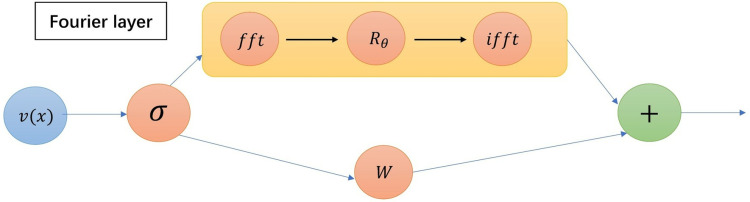
The Fourier layer: The internal structure of the Fourier layer.


Removing the dependence on a and supposing k(x,y;θ)=k(x−y;θ), the ∫Dk(x−y;θ)v(y)dy become a convolution operator. Then we directly parameterize the k(x,y;θ) in Fourier space, the 𝒦(a;θ) becomes the following Fourier integral operator, as shown in Fig 1:

$$(𝒦(a;\theta )v)(x)={ℱ}^{-1}(R_{\theta }\cdot F(v))(x),\forall x\in D.$$

Here, $R_{\theta }$ is related to the k(x,y;θ) via the Fourier transform, that is $R_{\theta }=F\left(k(x,y;\theta )\right)$. Apparently, ℱ is the Fourier transform and ${ℱ}^{-1}$ is the inverse Fourier transform:


$$ℱ\left[f(x)\right](k)=\int Df(x)e^{-2\pi i\left\langle x,k\right\rangle }dx,{ℱ}^{-1}\left[f(k)\right](x)=\int Df(k)e^{2\pi i\left\langle x,k\right\rangle }dk.$$

In our actual computation, we can use the Fast Fourier Transform (FFT) and Inverse Fast Fourier Transform (IFFT) to efficiently compute.

**Fig 2:**
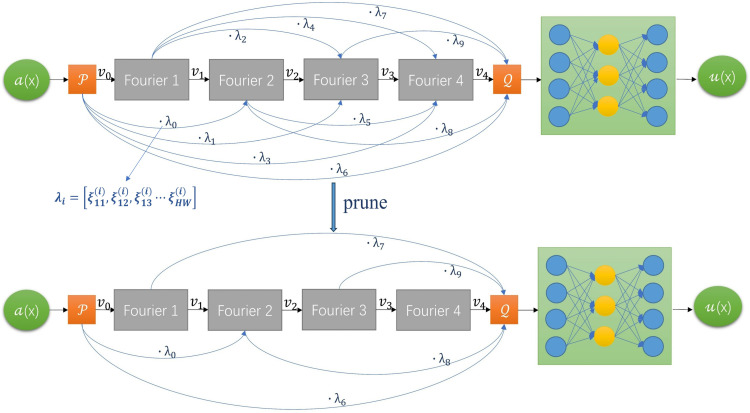
Process of pruning: First, pretrained above network framework for findingλ values. Use data in source domain ${𝒟}_{s}={\left\{\left(x_{i}^{s},y_{i}^{s}\right)\right\}}_{i=1}^{n_{s}}$ pre-train the network to get the optimal λ value. Then use the pruning method we proposed to prune the network, cut off the λ parameters that do not contribute much and select the λ parameters that need to be retained. This sparse structure can make our network faster and more compact without losing accuracy. The network below the picture is the pruned network, and we will perform subsequent transfer tasks on this network.

In this part, we explain how to get the value of λ and filter λ to complete the pruning of the network. In our network, λ is randomly initialized by the network to generate. Then, after the construction of the network framework, λ will be pre-trained with the network, as shown in Fig 2. It should be noted that we need to set a larger learning rate for λ parameter separately, to find its optimal solution in a larger domain. After the pre-training is completed, we get the optimal value of λ. From now on, we fix λ to the optimal value obtained after pre-training, so that, we can treat λ as a constant matrix. The network currently is a special dense net [34]. Next, we transform the dense net into an adaptive residual structure (residual structure is used in almost all neural network models) to improve its performance and efficiency, we need to use a reasonable mathematical method to prune the network architecture, i.e., to filter the constant matrix λ.

Assume there are $\lambda_{0},\lambda_{1}\cdots \lambda_{T}$ in our pre-trained network. According to (2), each λ is a matrix of size H×W. We flatten all λ into a row vector, and then form a matrix X:


$$X=(\begin{array}{c} \lambda_{0}\\ \begin{array}{c} \lambda_{1}\\ \begin{array}{c} \vdots \\ \lambda_{T} \end{array} \end{array} \end{array})=(\begin{array}{ccc} \begin{array}{ccc} \xi_{11}^{\left(0\right)} & \xi_{12}^{\left(0\right)} & \ldots \end{array} & \xi_{H,W-1}^{\left(0\right)} & \xi_{HW}^{\left(0\right)}\\ \begin{array}{ccc} \xi_{11}^{\left(1\right)} & \xi_{12}^{\left(1\right)} & \ldots \end{array} & \xi_{H,W-1}^{\left(1\right)} & \xi_{HW}^{\left(1\right)}\\ \begin{array}{ccc} \begin{array}{c} \vdots \\ \xi_{11}^{\left(T\right)} \end{array} & \begin{array}{c} \vdots \\ \xi_{12}^{\left(T\right)} \end{array} & \begin{array}{c} \ddots \\ \ldots \end{array} \end{array} & \begin{array}{c} \vdots \\ \xi_{H,W-1}^{\left(T\right)} \end{array} & \begin{array}{c} \vdots \\ \xi_{HW}^{\left(T\right)} \end{array} \end{array})\in {\mathbb{R}}^{T\times HW}.$$

Next, we find the covariance matrix D(X) of X:


$$D(X)=(\begin{array}{ccc} Cov\left(\lambda_{0},\lambda_{0}\right) & \cdots & Cov\left(\lambda_{0},\lambda_{T}\right)\\ \vdots & \ddots & \vdots \\ Cov\left(\lambda_{T},\lambda_{0}\right) & \cdots & Cov\left(\lambda_{T},\lambda_{T}\right) \end{array})\in {\mathbb{R}}^{T\times T},$$

since D(X) is a real symmetric matrix, it must be orthogonal to diagonalization:


$$D(X)=\Upsilon {(\begin{array}{ccc} k_{0} & & \\ & \ddots & \\ & & k_{T} \end{array})\Upsilon }^{T},$$

where the $k_{0}\cdots k_{T}$ are the eigenvalues of the matrix D(X), here we perform a simple sorting of the eigenvalues so that $k_{0}>k_{1}>\cdots >k_{T}$. The $\Upsilon \in {\mathbb{R}}^{T\times T}$ is a matrix composed of mutually orthogonal unit eigenvectors. Then we use the eigenvalues to determine how many λ we want to leave. There must be an integer s∈ℕ such that:


$$\frac{\sum_{i=0}^{s}k_{i}}{\sum_{j=0}^{T}k_{j}}\geq THand\frac{\sum_{i=0}^{s-1}k_{i}}{\sum_{j=0}^{T}k_{j}}<TH$$ (3)

where TH is a hyperparameter that needs to be set before training. The setting of this parameter depends on the degree of pruning you want, and it is set to 0.85 in subsequent experiments.

We take out the eigenvectors ${𝓅}_{0},{𝓅}_{1}\cdots {𝓅}_{s}$ corresponding to the eigenvalues $k_{0},k_{1}\cdots k_{s}$ from the $\Upsilon^{T}$ and sum them:


$$p=\sum_{i=0}^{s}{𝓅}_{i}=(\varphi_{0},\varphi_{1},\varphi_{2}\cdots \cdots \varphi_{T})\in {\mathbb{R}}^{T}.$$

Assume $\varphi_{0}<\varphi_{1}<\varphi_{2}<\cdots <\varphi_{T}$, then in the end, we keep the value of $\lambda_{T},\lambda_{T-1}\cdots \lambda_{T-s+1}$, i.e., one-to-one correspondence with the subscripts of the larger s elements in $\left(\varphi_{0},\varphi_{1},\varphi_{2}\cdots \cdots \varphi_{T}\right)$. Let the remaining $\lambda_{0}=\lambda_{1}=\cdots =\lambda_{T-s}=0$. Using the above algorithm, we complete the prune operation. The pruned network is shown in Fig 2. Subsequent transfer tasks are performed on the pruned network framework.

## 3. Transfer learning method

In this part, we mainly consider that there is enough labeled data in the source domain ${𝒟}_{s}={\left\{\left(x_{i}^{s},y_{i}^{s}\right)\right\}}_{i=1}^{n_{s}}$, where the vector of input random variables $x_{i}^{s}\in X_{s}$ and the corresponding output vector $y_{i}^{s}\in Y_{s}$. Furthermore, there is a target domain with many unlabeled data and few labeled data ${𝒟}_{t}={\left\{\left(x_{i}^{tl},y_{i}^{tl}\right)\right\}}_{i=1}^{n_{tl}}\cup {\left\{\left(x_{j}^{tu},?\right)\right\}}_{j=1}^{n_{tu}}$, where $x_{i}^{tl}$, $x_{j}^{tu}\in X_{s}$ and $y_{i}^{tl}\in Y_{t}$. Obviously, there are identical marginal distribution ($X_{s}$) and different conditional distribution ($Y_{s}$). After given the target domain and the source domain, transfer learning methods can be uniformly characterized as:


$$f_{t}=\mathrm{\backslash arg}\min\frac{1}{n_{s}}\sum_{i=1}^{ns}L(f_{s}(x_{i}^{s}),y_{i}^{s})+\alpha R({𝒟}_{t},f_{s}),$$

where the $R({𝒟}_{t},f_{s})$ is transfer regex.

Since we are using the pre-training and fine-tuning transfer method, which fine-tuning is a standard empirical risk minimization process, we need to add transfer regex to improve performance. In our experiments, we use the L2-SP regularization constraint in the fine-tuning:


$$R(w)=\alpha {\left\|w-w_{0}\right\|}_{2}^{2},$$

where the $w_{0}$ represents the starting point of weight. Note that the source and target network frameworks may differ slightly. In the common part (or with the same structure) we directly apply the above formula. In the other part, ordinary L2 regularization is used for constraints.

The construction of the transfer learning framework is based on λ-FNO. Since the subsequent experimental data is generated based on the unstructured grid, we put a fully connected layer at the end of the network to finally map the discrete input function on the structured grid to the discrete solution function on the unstructured grid.

### 3.1. Steps of the proposed method

#### Pre-training λ-FNO on source domain.

Dense λ-FNO (Fig 2) is pre-trained on a source domain ${𝒟}_{s}={\left\{\left(x_{i}^{s},y_{i}^{s}\right)\right\}}_{i=1}^{n_{s}}$, where $x_{i}^{s}\in X_{s}$, $y_{i}^{s}\in Y_{s}$. We pre-training the source model using the relative L2 error:


$$ℒ(\theta )=\frac{{\left\|f_{s}(x^{s})-y^{s}\right\|}_{2}}{{\left\|y^{s}\right\|}_{2}},$$

where ${\left\|\cdot \right\|}_{2}$ is Euclidean norm. $f_{s}(x^{s})$ and $y^{s}$ are prediction data and label data respectively. In the optimization part, we adopt the Adam optimizer to minimize the loss function. After the training is completed, we need to save the λ parameter.

#### Pruning the network.

Filter the λ parameter through the algorithm in part 2 to complete the pruning of the network. We fix the value of λ that passed the screening to make it a constant matrix and set it to a zero matrix if it failed the screening. The pruned network is shown in Fig 2.

#### Parameters transfer.

Transfer the training parameters of λ-FNO to the target model TL-λFNO. This method is very commonly used in transfer learning, which can effectively reduce the dependence on the label data of the target domain and reduce the cost of training from scratch. TL-λFNO remains roughly the same, only a slight change to the output layer neurons of the last fully connected layer is required.

#### Fine-tuning TL-λFNO on target domain.

**Fig 3:**
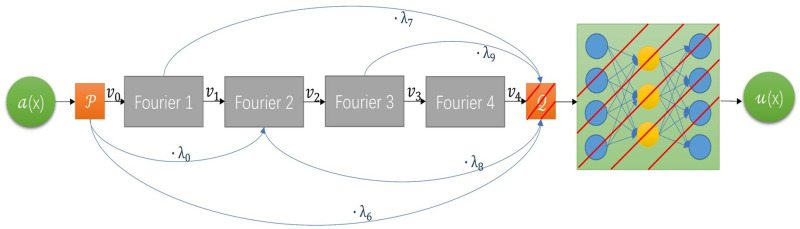
Target model (TL-λFNO): Transfer the parameters trained on the source model to the target model. Except for the dimensionality reduction operator 𝒬 (including two fully connected layers inside) and the last fully connected layer (red shaded part), other layer parameters are frozen. The network layers represented by the red shaded part are fine-tuned using the labeled data in target domain ${𝒟}_{t}={\left\{\left(x_{i}^{tl},y_{i}^{tl}\right)\right\}}_{i=1}^{n_{tl}}\cup {\left\{\left(x_{j}^{tu},?\right)\right\}}_{j=1}^{n_{tu}}$ and hybrid loss function $ℒ\left(\theta^{T}\right)$.


Fine-tuning some specific layers of TL-λFNO in the target domain ${𝒟}_{t}={\left\{\left(x_{i}^{tl},y_{i}^{tl}\right)\right\}}_{i=1}^{n_{tl}}\cup {\left\{\left(x_{j}^{tu},?\right)\right\}}_{j=1}^{n_{tu}}$. In the field of computer vision, it is generally believed that the parameters of the convolutional layer are general, while the parameters of the fully connected layer are used for specific task goals [35–36]. So, we freeze the parameters of all layers except the last three layers of fully connected layers, and then fine-tune the parameters of the last three layers through the hybrid loss function (fine-tune the parameters of the red shaded part of Fig 3). Ensure that it still has excellent results for specific tasks while reducing training costs and reducing the occurrence of overfitting. The transfer regex in the hybrid loss function is conducive to the closeness of the parameter’s distribution of the target domain model and the source domain model, which can effectively improve the transfer performance, and reduce the overfitting of the model. The hybrid loss function define as follows:

$$ℒ(\theta^{T})=\frac{{\left\|f_{TL}(x^{tl})-y^{tl}\right\|}_{2}}{{\left\|y^{tl}\right\|}_{2}}+{\alpha \left\|w-w_{0}\right\|}_{2}^{2},$$

where α is an adjustable hyper-parameter that was set to 0.0001 in subsequent experiments. w and $w_{0}$ denote the current value and initial value of the target model parameters (i.e., transfer initial value) respectively. In the optimization process, we still use the Adam optimizer to minimize the loss and finally get the optimal parameters.

## 4. Numerical experiments

In this section, we performed some specific transfer tasks for Darcy Flow, Elasticity model, and Burgers’ equation. All tasks consider transfer learning under conditional shift, that is, the distribution of task input data is the same but there is a difference in output distribution. All experiments uniformly use four Fourier operator layers, ReLU activation function and Adam optimizer. The error item in the table is the average value of the test set error obtained in the last 10 epochs. All calculations are performed on a single NVIDIA GeForce RTX 3,060 Mobile GPU.

### 4.1. Darcy Flow

The 2-d Darcy Flow equation which is a linear second-order PDE on the unit square box is described by:


$$\nabla \cdot (𝒶(x)\nabla 𝓊(x))=g(x),x=(x_{1},x_{2})\in [0,1{]}^{2},$$

$$𝓊(x)=0,x\in \partial [0,1{]}^{2},$$

where 𝒶(x) is the diffusion coefficient and g(x) is the forcing function. For simplicity, we directly consider a constant forcing term, i.e., g(x)=1. We uniformly impose the Dirichlet boundary condition on the boundary. This PDE can be applied in industrial fields such as the control of groundwater pollution and the exploitation of fracture-cavity reservoirs. We want to learn the operator which nonlinearly maps the diffusion coefficient 𝒶(x) to the solution 𝓊(x), i.e., ${𝒢}_{\theta }:a\to u$.

**Fig 4:**
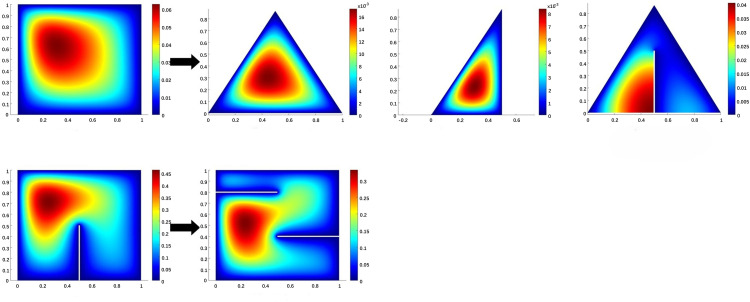
Transfer learning for Darcy Flow: Geometry differences between source and target domains in TL1-TL4.

For this PDE, we consider the following four transfer tasks which are presented in Fig 4:

1. TL1: Transfer from the square domain to the equilateral triangle domain.
2. TL2: Transfer from a square domain to a right-angled triangle.
3. TL3: Transfer from a square domain to an equilateral triangle with a vertical notch.
4. TL4: Transfer from a square domain with one vertical notch to a square domain with two horizontal notches.

To train the operator network, we randomly sample 𝒶(x) and generate the corresponding model response 𝓊(x). For source domain data, we uniformly generate $N_{s}=2000$ training data and $N_{s}^{test}=100$ test data. In addition, for the target domain, we also generate $N_{t}=2000$ training data and $N_{t}^{test}=100$ test data. When training on the target domain, sequential training is performed for $N_{t}$ =  {5, 20, 50, 100, 150, 200, 250, 2,000} samples and $N_{t}^{test}=100$ test data to evaluate the effect of training set size on the network impact. We train on the source domain using DeepONet, FNO, and our proposed λ-FNO, respectively. The relative L2 norm error (%) and training cost (s) are shown in Tables 1, 5 and 7 respectively. In terms of source domain accuracy, λ-FNO has an absolute advantage over DeepONet and FNO. The training speed is only slightly slower than FNO and faster than DeepONet. The Training cost of DeepONet can be obtained in reference [31]. For the target domain, we construct TL-λFNO for TL1–4. On the one hand, we train the target domain data on a new λ-FNO from scratch. On the other hand, we fine-tune the target domain data on TL-λFNO which is constructed by transfer of source domain network parameters. The target domain relative L2 norm error (%) and training cost (s) of TL1–4 are in Tables 2–4, 6 and 8 respectively. Under the extremely small data of $N_{t}=5$, TL-DeepOnet has a better effect. When $N_{t}$ =  {50, 100, 150, 200, 250, 2000}, TL-λFNO has a huge advantage over training on TL-DeepONet. The reason is that TL-λFNO contains a lot of source domain information, guaranteed very good accuracy even when little data is available. So, fine-tuning TL-λFNO is more correct than retraining on λ-FNO. In general, the lower bound of the downstream transfer task is tightly dependent on the performance of pre-training. So, it is only natural that TL-λFNO outperforms TL-DeepONet in most cases. In the case of a large amount of data, such as $N_{t}=2000$, training λ-FNO from scratch can achieve the highest accuracy, but at the same time there will be a high time cost. As shown in Table 8, when $N_{t}=2000$, the time to train λ-FNO from scratch is 4,102, 4,128, 4,073 and 3,983 seconds respectively. If the accuracy requirements are not strict, fine-tuning TL-λFNO is still the best choice. When $N_{t}=2000$, the relative L2 norm error of fine-tuning TL-λFNO is 0.51%, 0.73%, 0.98% and 0.33% for TL1–4. The training time is only 654, 640, 666 and 637 seconds respectively, which takes less than a quarter of the time compared to training λ-FNO from scratch. This is expected, since we only need to train its last three fully connected layers when fine-tuning TL-λFNO, which can greatly reduce training parameters and time. However, the parameters transferred from the source model contain enough information to ensure accuracy. Through this result, we can also judge that when transfer learning is applied to Darcy Flow, it can reduce the cost to a certain extent, whether it is in terms of time cost or data cost.

**Table 1:** Relative L2 error (%) on the test set for the source domain (TL1 – TL3).

TL1–3(Source)			
N	DeepONet	FNO	λ-FNO
2000	1.36 ± 0.48	0.30 ± 0.02	**0.20 ± 0.02**

**Table 2:** Relative L2 error (%) on the test set for the target domain (TL1).

TL1(Target)				
N	DeepONet	λ-FNO	TL-DeepONet	TL-λFNO
5	31.60 ± 0.36	55.25 ± 1.86	**10.70 ± 0.53**	31.20 ± 0.91
20	26.50 ± 0.62	15.22 ± 1.44	8.81 ± 0.23	**8.53 ± 0.12**
50	26.41 ± 0.54	10.12 ± 0.87	7.37 ± 0.18	**3.88 ± 0.27**
100	19.20 ± 0.36	4.82 ± 1.03	6.51 ± 0.28	**2.22 ± 0.30**
150	14.40 ± 0.33	2.83 ± 0.28	5.61 ± 0.30	**1.65 ± 0.04**
200	11.90 ± 0.32	2.22 ± 0.40	3.85 ± 0.07	**1.43 ± 0.03**
250	9.50 ± 0.10	2.16 ± 0.19	3.74 ± 0.07	**1.27 ± 0.08**
2000	1.40 ± 0.10	**0.19 ± 0.02**	3.19 ± 0.14	0.51 ± 0.02

**Table 3:** Relative L2 error (%) on the test set for the target domain (TL2).

TL2(Target)				
N	DeepONet	λ-FNO	TL-DeepONet	TL-λFNO
5	25.53 ± 1.41	53.34 ± 2.86	**11.73 ± 1.59**	28.38 ± 0.89
20	19.68 ± 0.39	19.82 ± 0.84	**8.79 ± 0.30**	11.66 ± 0.19
50	16.68 ± 0.34	9.85 ± 2.37	7.42 ± 0.48	**6.22 ± 0.25**
100	15.39 ± 0.26	3.82 ± 0.73	6.51 ± 0.28	**2.90 ± 0.28**
150	13.20 ± 0.22	2.19 ± 0.06	5.54 ± 0.33	**2.05 ± 0.25**
200	12.03 ± 0.09	**1.74 ± 0.40**	4.44 ± 0.32	1.87 ± 0.13
250	8.32 ± 0.09	1.52 ± 0.21	3.46 ± 0.27	**1.51 ± 0.05**
2000	1.62 ± 0.30	**0.15 ± 0.02**	2.19 ± 0.17	0.73 ± 0.02

**Table 4:** Relative L2 error (%) on the test set for the target domain (TL3).

TL3(Target)				
N	DeepONet	λ-FNO	TL-DeepONet	TL-λFNO
5	29.14 ± 0.52	46.94 ± 1.02	**12.28 ± 0.44**	26.88 ± 1.57
20	25.57 ± 0.43	16.52 ± 1.34	**9.54 ± 0.05**	11.86 ± 1.58
50	18.68 ± 0.36	10.85 ± 1.25	7.54 ± 0.76	**5.82 ± 0.19**
100	15.56 ± 0.34	5.02 ± 0.31	6.45 ± 0.36	**3.20 ± 0.08**
150	14.71 ± 0.30	3.07 ± 0.30	6.04 ± 0.28	**2.51 ± 0.11**
200	12.32 ± 0.29	2.26 ± 0.15	5.28 ± 0.19	**2.14 ± 0.08**
250	11.75 ± 0.22	2.00 ± 0.38	4.92 ± 0.15	**1.95 ± 0.06**
2000	1.83 ± 0.37	**0.19 ± 0.02**	4.72 ± 0.22	0.98 ± 0.06

**Table 5:** Relative L2 error (%) on the test set for the source domain (TL4).

TL4(Source)			
N	DeepONet	FNO	λ-FNO
2000	1.58 ± 0.05	0.28 ± 0.01	**0.19 ± 0.02**

**Table 6:** Relative L2 error (%) on the test set for the target domain (TL4).

TL4(Target)				
N	DeepONet	λ-FNO	TL-DeepONet	TL-λFNO
5	51.70 ± 0.18	51.04 ± 0.72	**10.46 ± 0.93**	21.27 ± 0.92
20	39.69 ± 0.13	14.92 ± 1.14	8.16 ± 0.29	**8.06 ± 0.78**
50	25.82 ± 0.62	8.45 ± 0.79	7.01 ± 0.17	**3.32 ± 0.22**
100	19.50 ± 0.25	5.35 ± 0.51	5.78 ± 0.42	**1.90 ± 0.13**
150	16.82 ± 0.23	2.97 ± 0.12	5.10 ± 0.15	**1.19 ± 0.08**
200	12.00 ± 0.27	2.01 ± 0.29	4.11 ± 0.26	**0.85 ± 0.15**
250	9.22 ± 0.04	1.67 ± 0.15	3.34 ± 0.09	**0.78 ± 0.06**
2000	1.78 ± 0.18	**0.18 ± 0.01**	2.14 ± 0.03	0.33 ± 0.04

**Table 7:** Training cost in seconds (s) for the source domain (TL1-3 and TL4).

Source	FNO	λ-FNO
TL1–3 source	3,741	4,175
TL4 source	3,730	4,131

**Table 8:** Training cost in seconds (s) for the target domain (TL1-TL4).

Target N		TL1	TL2	TL3	TL4
Training λ-FNO (Target)	5	81	81	83	78
	20	246	248	255	248
	50	565	582	572	558
	100	585	585	565	559
	150	594	592	595	579
	200	614	632	634	627
	250	620	648	632	638
	2000	4,102	4,128	4,073	3,983
Training TL-λFNO (Target)	5	**31**	**29**	**31**	**30**
	20	**120**	**116**	**119**	**120**
	50	**280**	**260**	**263**	**255**
	100	**259**	**257**	**264**	**253**
	150	**263**	**259**	**262**	**254**
	200	**267**	**265**	**265**	**261**
	250	**265**	**272**	**274**	**276**
	2000	**654**	**640**	**666**	**637**

### 4.2 .Elasticity model

We consider modeling a thin rectangular plate subjected to in-plane loading as a two-dimensional plane stress elasticity problem:


∇·σ+f(x)=0,x=(x,y)

(u,v)=0,x=0

where σ is the Cauchy stress tensor, f(x) is the body force, u(x) represents the x-displacement and v(x) represents the y-displacement. The relation between displacement and stress under plane stress is defined as:


$$\left\{\begin{array}{c} \sigma_{xx}\\ \sigma_{yy}\\ \tau_{xy} \end{array}\right\}=\frac{E}{1-\mu^{2}}[\begin{array}{ccc} 1 & \mu & 0\\ \mu & 1 & 0\\ 0 & 0 & \frac{1-\mu }{2} \end{array}]\times \left\{\begin{array}{c} \varepsilon_{x}\\ \varepsilon_{y}\\ \gamma_{xy} \end{array}\right\},$$

$$\varepsilon_{x}=\frac{\partial u}{\partial x},\varepsilon_{y}=\frac{\partial v}{\partial y},\gamma_{xy}=\frac{\partial u}{\partial y}+\frac{\partial v}{\partial x},$$

where E represents Young’s Modulus and μ represents Poisson’s Ratio.

For the elasticity model, what we want to learn is the mapping operator from random boundary loads to displacement fields (u: x-displacement and v: y-displacement), i.e., ${𝒢}_{\theta }:f(x)\to \left[u\left(x\right),v(x)\right]$. We still choose to randomly sample f(x) from a Gaussian random field. For the source domain, we consider a thin plate with a centered circular internal boundary and material properties ($E_{s}=300\times 10^{5},\mu_{s}=0.3$). Furthermore, based on different geometric shapes, in this application we will consider that the source domain and the target domain have different material properties. This not only increases the difficulty of our network framework, but also demonstrates the wide applicability and practical application value of transfer learning.

**Fig 5:**
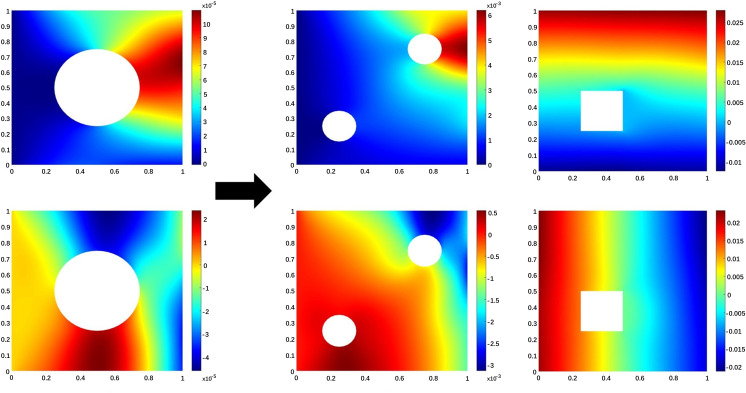
Transfer learning for Elasticity model: Geometry differences between source and target domains in TL5-TL6.

For this experiment, we consider the following four transfer tasks which are presented in Fig 5:

1. TL5: Transfer the source domain with a centered circular inner boundary and material properties ($E_{s}=300\times 10^{5},\mu_{s}=0.3$) to two smaller circular inner boundaries with upper right and lower left corners and different material properties ($E_{t}=410\times 10^{3},\mu_{t}=0.35$) of the target domain.
2. TL6: Transfer a source domain with a centered circular interior boundary and material properties ($E_{s}=300\times 10^{5},\mu_{s}=0.3$) to a target domain with a small square interior boundary and different material properties ($E_{t}=410\times 10^{3},\mu_{t}=0.45$).

To train the source domain model, we randomly sample N=2000 random boundary loads functions f(x) and get the corresponding displacements u(x) and v(x). Randomly divide 2,000 data into $N_{s}=1900$ training data and $N_{s}^{test}=100$ test data. We still compare DeepONet, FNO and our proposed λ-FNO framework, the results of the source domain are shown in Table 9 and Table 10. From the analysis of the results, the proposed λ-FNO is still superior in accuracy, and only slightly inferior to FNO in speed. This proves that our proposed framework is effective. For the target domain, we still sample $N_{t}$ =  {5, 20, 50, 100, 150, 200, 250, 1900} training data and $N_{t}^{test}=100$ test data for sequential training and evaluation. So that we can observe whether there is an advantage in fine-tuning the transferred model compared to training our network from scratch when there is only a small amount of data in the target domain. All relative L2 error (%) results for TL5 and TL6 are shown in Table 11 and 12, respectively. From the result analysis, in the case of extremely small data such as $N_{t}=5$, fine-tuning TL-DeepONet still performs better. When the number of training data samples is $N_{t}$ =  {20, 50, 100, 150, 200, 250, 1900}, compared to training λ-FNO from scratch, fine-tuning our transfer model TL-λFNO can achieve smaller errors and greatly improve the prediction accuracy of our model. Moreover, the error of our proposed TL-λFNO is reduced to another order of magnitude compared to the transfer model TL-DeepONet, i.e., For TL5 target domain, when $N_{t}=250$, the test errors of TL-DeepONet are 3.56% and 4.33% respectively, while the test errors of TL-λFNO are only 0.38% and 0.84%. In the case that the target domain itself has a large amount of data, training the network framework from scratch will lead to smaller errors, which is expected. We observe that for $N_{t}=1900$ the resulting TL-λFNO accuracy is like the case of training λ-FNO on the target domain but the time cost is expensive for training from scratch, as shown in Table 13. Therefore, when the accuracy requirements are not very strict or the amount of data in the target domain is small, it is very practical to use transfer learning to reduce time costs and improve accuracy.

**Table 9:** Relative L2 error (%) on the test set for the source domain (TL5 – TL6).

N	DeepONet		FNO		λ-FNO	
u(x)	v(x)	u(x)	v(x)	u(x)	v(x)
1900	2.26 ± 0.09	2.52 ± 0.18	0.43 ± 0.01	0.58 ± 0.01	**0.15 ± 0.01**	**0.21 ± 0.01**

**Table 10:** Training cost in seconds (s) for the source domain (TL5-6).

Source	FNO	λ-FNO
TL5–6source	2,401	2,637

**Table 11:** Relative L2 error (%) on the test set for the target domain (TL5).

	N	DeepONet		λ-FNO	
u(x)	v(x)	u(x)	v(x)
Training DeepONet and λ-FNO directly (Target)	5	198.36 ± 34.4	296.0 ± 45.90	68.11 ± 2.45	125.22 ± 2.53
	20	67.70 ± 12.30	91.68 ± 30.81	21.80 ± 0.39	35.45 ± 1.54
	50	21.90 ± 3.36	24.3 ± 5.16	5.02 ± 0.41	8.52 ± 1.41
	100	11.6 ± 3.59	13.26 ± 3.07	1.84 ± 0.11	3.54 ± 0.44
	150	8.42 ± 0.81	12.34 ± 3.41	1.25 ± 0.14	2.23 ± 0.45
	200	7.34 ± 0.05	9.18 ± 1.80	0.89 ± 0.10	1.87 ± 0.63
	250	5.98 ± 0.05	7.16 ± 0.86	0.75 ± 0.06	1.53 ± 0.20
	1900	1.72 ± 0.08	2.80 ± 0.25	**0.11 ± 0.01**	**0.20 ± 0.01**
	N	TL-DeepONet		TL-λFNO	
		u(x)	v(x)	u(x)	v(x)
Training TL-DeepONet and TL-λFNO (Target)	5	**8.17 ± 0.05**	**11.31 ± 1.36**	17.07 ± 0.09	42.96 ± 1.21
	20	5.94 ± 0.33	10.7 ± 0.27	**2.38 ± 0.15**	**5.76 ± 0.94**
	50	4.72 ± 0.44	9.84 ± 0.05	**0.90 ± 0.11**	**2.03 ± 0.62**
	100	4.42 ± 0.24	8.58 ± 0.15	**0.52 ± 0.15**	**1.12 ± 0.22**
	150	4.14 ± 0.05	7.2 ± 0.01	**0.45 ± 0.06**	**1.02 ± 0.30**
	200	3.84 ± 0.05	6.11 ± 0.02	**0.40 ± 0.08**	**0.95 ± 0.23**
	250	3.56 ± 0.05	4.33 ± 0.05	**0.38 ± 0.04**	**0.84 ± 0.16**
	1900	2.50 ± 0.01	2.84 ± 0.05	0.27 ± 0.01	0.58 ± 0.05

**Table 12:** Relative L2 error (%) on the test set for the target domain (TL6).

	N	DeepONet		λ-FNO	
u(x)	v(x)	u(x)	v(x)
Training DeepONet and λ-FNO directly (Target)	5	137.5 ± 31.15	171.9 ± 24.07	125.96 ± 7.32	173.24 ± 6.35
	20	131.36 ± 83.07	149.66 ± 54.49	36.23 ± 2.18	47.94 ± 2.56
	50	28.14 ± 8.3	27.8 ± 7.43	9.03 ± 1.01	16.87 ± 1.95
	100	10.62 ± 1.64	11.44 ± 1.38	3.15 ± 0.18	4.47 ± 0.58
	150	8.76 ± 1.67	11.02 ± 1.38	1.89 ± 0.25	3.82 ± 1.24
	200	8.93 ± 1.32	10.93 ± 1.35	1.82 ± 0.19	2.73 ± 0.25
	250	9.14 ± 0.60	10.7 ± 1.41	1.46 ± 0.28	2.05 ± 0.38
	1900	2.72 ± 0.26	1.92 ± 0.41	**0.18 ± 0.01**	**0.25 ± 0.03**
	N	TL-DeepONet		TL-λFNO	
		u(x)	v(x)	u(x)	v(x)
Training TL-DeepONet and TL-λFNO (Target)	5	281.9 ± 1.15	178.2 ± 1.36	**37.16 ± 2.23**	**63.34 ± 3.17**
	20	17.30 ± 0.19	23.90 ± 0.17	**5.05 ± 1.11**	**7.93 ± 1.52**
	50	16.01 ± 0.12	15.11 ± 0.15	**1.62 ± 0.35**	**2.64 ± 0.42**
	100	15.04 ± 0.13	12.50 ± 0.15	**0.83 ± 0.18**	**1.31 ± 0.20**
	150	13.14 ± 0.05	11.97 ± 0.09	**0.75 ± 0.13**	**1.17 ± 0.32**
	200	12.43 ± 0.05	11.32 ± 0.12	**0.69 ± 0.08**	**1.02 ± 0.17**
	250	11.12 ± 0.04	9.66 ± 0.14	**0.58 ± 0.05**	**0.99 ± 0.12**
	1900	7.62 ± 0.04	6.1 ± 0.10	0.44 ± 0.01	0.61 ± 0.04

**Table 13:** Training cost in seconds (s) for the target domain (TL5 and TL6).

Target	TL5		TL6	
N	λ-FNO	TL-λFNO	λ-FNO	TL-λFNO
5	69	**41**	72	**57**
20	342	**205**	349	**219**
50	1,046	**631**	1,114	**641**
100	1,351	**782**	1,402	**837**
150	1,330	**788**	1,374	**816**
200	1,390	**797**	1,333	**835**
250	1,420	**789**	1,369	**816**
1900	2,587	**827**	2,616	**864**

### 4.3 .Burgers’ equation

We consider the 1-d Burgers’ equation which is a nonlinear parabolic equation. It takes the form


$$\partial_{t}u(x,t)+\partial_{x}\left(\frac{u^{2}(x,t)}{2}\right)=\upsilon \partial_{xx}u(x,t),x\in (0,1),t\in (0,1],$$

$$u(x,0)=u_{0}(x),x\in (0,1),$$

where u(x,t) represents the velocity field of the fluid with periodic boundary conditions and υ>0 is the diffusion coefficient. The Burgers’ equation is a fundamental partial differential equation in various fields of applied mathematics, such as fluid mechanics, nonlinear acoustics, gas dynamics. We aim to learn the operator mapping ${𝒢}_{\theta }$ between the initial condition $u(x,t=0)=u_{0}(x)$ and the equation’ solution at time one u(x,t=1). The initial condition $u_{0}(x)$ is randomly sampled from a Gaussian random field. For the source model, we choose the diffusion coefficient υ=0.2 for the equation.

For Burgers’ equation, we consider the following transfer scenario:

1.TL7: Transfer learning from diffusion coefficients $\upsilon_{1}=0.2$ to $\upsilon_{2}=0.1$.

**Fig 6:**
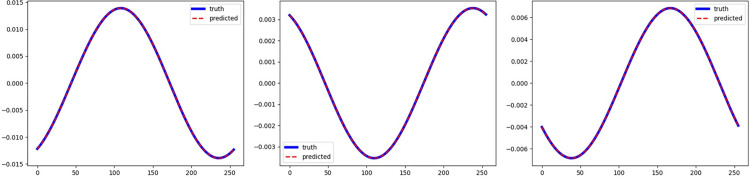
Plots of three representative realizations of the initial condition with reference response.

For the source model, we generate $N_{s}=2000$ train and $N_{s}^{test}=200$ test data. In addition, we generate $N_{t}=500$, $N_{t}^{test}=200$ train and test target data. When training the target model, we sample $N_{t}$ =  {5, 10, 15, 20, 25, 50, 100, 500} training data to train the model in sequence and use $N_{t}^{test}=200$ test data to test the target model. It is worth noting that due to the small proportion of the last three layers of network parameters in this experiment, the last Fourier layer was added to the fine-tuning process during the fine-tuning transfer model. The relative L2 error (%) results of the test set on the source model are shown in Table 14. It can be seen from the Table 14 that even when the magnitude of the error is very small, if the training is extreme, the λ-FNO proposed by us can still reduce the error by more than 15%. For the target domain, we still sample $N_{t}$ =  {5, 10, 15, 20, 25, 50, 100, 500} training data and $N_{t}^{test}=200$ test data for sequential training and evaluation. We use two training modes, one is to fine-tune the TL-λFNO which is transferred from source model, and the other is to train a new λ-FNO network from scratch, the results are shown in Table 15. It can be seen from the table that when there is little data in the target domain, i.e., $N_{t}=5,20$, fine-tuning TL-λFNO can get higher accuracy. When the amount of data becomes larger, the accuracy obtained by training a new λ-FNO from scratch is gradually approaching the accuracy obtained by fine-tuning TL-λFNO. But in terms of time cost, it still takes less time to fine-tune TL-λFNO, as shown in Table 16. Overall, when the target domain data is scarce, the method using the fine-tuned transfer model turns out to be better both in terms of accuracy and time cost. When there is more data in the target domain, if you pursue higher accuracy, you should choose to train a new λ-FNO from scratch. If you pursue training speed, fine-tuning the transfer model is still a better choice. Finally, plots of three representative realizations of the initial condition with reference response are shown in Fig 6.

**Table 14:** Relative L2 error (%) on the test set for the source domain (TL7).

TL7(Source)		
N	FNO	λ-FNO
2000	0.097 ± 0.011	**0.075 ± 0.012**

**Table 15:** Relative L2 error (%) on the test set for the target domain (TL7).

	N	TL7(Target)	
λ-FNO	TL-λFNO
Training TL-λFNO and λ-FNO (Target)	5	19.73 ± 0.39	**4.55 ± 0.17**
	10	2.03 ± 0.14	**1.52 ± 0.22**
	15	0.98 ± 0.13	**0.92 ± 0.02**
	20	0.70 ± 0.07	**0.64 ± 0.02**
	25	**0.52 ± 0.07**	0.56 ± 0.05
	50	0.25 ± 0.03	**0.28 ± 0.01**
	100	0.13 ± 0.04	**0.11 ± 0.02**
	500	**0.043 ± 0.002**	0.069 ± 0.012

**Table 16:** Training cost in seconds (s) for the target domain (TL7).

Target	TL7	
N	λ-FNO	TL-λFNO
5	68	**46**
10	133	**81**
15	194	**152**
20	285	**198**
25	344	**258**
50	700	**554**
100	721	**568**
500	914	**549**

## 5. Discussion and conclusion

In conclusion, we successfully construct a new framework λ-FNO based on FNO by introducing λ parameter and pruning method. λ-FNO is then used for the transfer scenario of PDEs on unstructured grid discretization. We conduct numerical experiments on Darcy Flow, Elasticity model, and Burgers’ equation respectively. First, FNO and λ-FNO are trained and compared using source domain data. On the source model, the error we get with λ-FNO is about 20%−50% lower than that of FNO. Then use λ-FNO for transfer learning. We constructed a total of 7 transfer scenarios and used two training methods, one is to train λ-FNO from scratch using the target domain data, and the other is to fine-tune the TL-λFNO transferred from the source model. Experimental results show that using a good transfer model can effectively reduce data cost and time cost. Finally, we compared the error results of our proposed TL-λFNO with the TL-DeepONet proposed in the reference [31], and the results show that TL-λFNO has better performance in terms of accuracy and speed. It is worth noting that we believe that the λ-FNO construction method proposed in this paper, i.e., the introduction and pruning of λ parameters, can not only be applied to FNO but also can be extended to other networks. In our expectation, this method can achieve higher accuracy than ResNet and faster speed than DenseNet. We will have a more in-depth study on it later.

## Appendix A

### Data generation

In this section, we provide the parameters of data generator for the three equation we used in Section 4.

#### 1.1. Burgers’ equation.

The 1-d Burgers’ equation takes the form:


$$\partial_{t}u(x,t)+\partial_{x}\left(\frac{u^{2}(x,t)}{2}\right)=\upsilon \partial_{xx}u(x,t),x\in (0,1),t\in (0,1],$$

$$u(x,0)=u_{0}(x),x\in (0,1).$$

The initial conditions $u_{0}(x)$ are randomly sampled in a Gaussian random field from $u_{0}\sim \mu$, where $\mu =𝒩(0,49^{2}{\left(-\Delta +49I\right)}^{-2.5})$ with periodic boundary conditions. We uniformly take 128 points for $u_{0}(x)$ in the definition domain (0,1), and represent the initial condition $u_{0}(x)$ in a discretized form. Then solve the equations using a split step method, where the heat equation part is solved exactly in Fourier space, and then the nonlinear part is solved again in Fourier space using the forward Euler method. We solve on a spatial grid with resolution $2^{8}=256$.

#### 1.2. Darcy Flow.

Recall the 2-d Darcy Flow on the unit square box:


$$\nabla \cdot (𝒶(x)\nabla 𝓊(x))=g(x),x=(x_{1},x_{2})\in [0,1{]}^{2},$$

$$𝓊(x)=0,x\in \partial [0,1{]}^{2}.$$

The 𝒶(x) is described as a stochastic process and generated according to $a\sim GP(0,𝒦(x,x^{\prime }))$, where $𝒦\left(x,x^{\prime }\right)=exp\left[-\frac{{\left({x-x}^{\prime }\right)}^{2}}{2l^{2}}\right],l=0.25,x,x^{\prime }\in [0,1{]}^{2}$. We discretize 𝒶(x) in the 100×100 grid. The realizations are generated with a truncated Karhunen-Loéve expansion (KLE). The Dirichlet boundary conditions are imposed on all boundaries. For the TL1–3, We employ 1,541, 2,295, 1,200, 2,295 unstructured meshes in our simulations for the square, equilateral triangle, the right-angled triangle, and the triangular domain with a notch, respectively. For the TL4, we employ 1,538, 1,552 unstructured meshes in our simulations for the square domain with a vertical notch and the square domain with two horizontal notches, respectively.

#### 1.3. Elasticity model.

The rectangular plate subjected to in-plane loading as a two-dimensional plane stress elasticity problem:


∇·σ+f(x)=0,x=(x,y),

(u,v)=0,x=0.

The random boundary loads f(x) is described as a stochastic process and generated according to $f\sim GP\left(0,𝒦(x,x^{\prime })\right)$, where $𝒦(x,x^{\prime })=exp\left[-\frac{{\left(x-x^{\prime }\right)}^{2}}{2l^{2}}\right],l=0.12,x,x^{\prime }\in [0,1]$. We discretize f(x) in the 101 grid. The realizations are generated with a truncated Karhunen-Loéve expansion (KLE). For the TL5–6, we employ 1,020, 1,183, 1816 unstructured meshes in our simulations for the source domain, TL5 target domain and the TL6 target domain respectively.

## Appendix B

### Network and training parameters

We uniformly use 0.001 and 0.01 as the initial learning rate of network parameters and λ parameters respectively. In this section, we will give the necessary hyperparameters for each training task (Table 17).

**Table 17:** Training hyperparameter.

Scenario	Training number	Batch size	Epochs	Scheduler step
Training FNO on TL1–4 source	2000	50	2,500	500
Training λ-FNO on TL1–4 source	2000	50	2,500	500
Training TL-λFNO on TL1–4 target	5	1	400	400
	20	1	600	300
	50	1	600	200
	100	2	600	200
	150	3	600	200
	200	4	600	200
	250	5	600	200
	2000	50	1,000	250
Training λ-FNO on TL1–4 target	5	1	600	300
	20	1	600	300
	50	1	600	300
	100	2	600	200
	150	3	600	200
	200	4	600	200
	250	5	600	200
	2000	50	2,500	500
Training FNO on TL5–6 source	1900	100	8,000	1,000
Training λ-FNO on TL5–6 source	1900	100	8,000	1,000
Training TL-λFNO on TL5–6 target	5	1	800	400
	20	1	1,200	400
	50	1	1,600	400
	100	2	2000	400
	150	3	2000	400
	200	4	2000	400
	250	5	2000	400
	1900	50	2,400	400
Training λ-FNO on TL5–6 target	5	1	800	400
	20	1	1,200	400
	50	1	1,600	400
	100	2	2000	400
	150	3	2000	400
	200	4	2000	400
	250	5	2000	400
	1900	100	8,000	1,000
Training FNO on TL7 source	2000	50	3,000	250
Training λ-FNO on TL7 source	2000	50	3,000	250
Training TL-λFNO on TL7 target	5	1	1,000	150
	10	1	1,000	150
	15	1	1,300	150
	20	1	1,300	150
	25	1	1,300	150
	50	1	1,500	150
	100	2	1,500	150
	500	10	1,500	150
Training λ-FNO on TL7 target	5	1	1,000	150
	10	1	1,000	150
	15	1	1,000	150
	20	1	1,100	150
	25	1	1,100	150
	50	1	1,100	150
	100	2	1,200	150
	500	10	1,200	150
